# Cardiovascular Disease Prevention: The Earlier the Better? A Review of Plant Sterol Metabolism and Implications of Childhood Supplementation

**DOI:** 10.3390/ijms21010128

**Published:** 2019-12-24

**Authors:** Bianca Scolaro, Leticia F.S. de Andrade, Inar A. Castro

**Affiliations:** Department of Food and Experimental Nutrition, Faculty of Pharmaceutical Sciences, University of São Paulo, 05508-000 São Paulo, Brazil; biancascolaro@gmail.com (B.S.); lefernanda.andrade@gmail.com (L.F.S.d.A.)

**Keywords:** Atherosclerosis, plant sterol, cholesterol-lowering, diet

## Abstract

Atherosclerosis is the underlying cause of major cardiovascular events. The development of atherosclerotic plaques begins early in life, indicating that dietary interventions in childhood might be more effective at preventing cardiovascular disease (CVD) than treating established CVD in adulthood. Although plant sterols are considered safe and consistently effective in lowering plasma cholesterol, the health effects of early-life supplementation are unclear. Studies suggest there is an age-dependent effect on plant sterol metabolism: at a younger age, plant sterol absorption might be increased, while esterification and elimination might be decreased. Worryingly, the introduction of low-cholesterol diets in childhood may unintentionally favor a higher intake of plant sterols. Although CVD prevention should start as early as possible, more studies are needed to better elucidate the long-term effects of plant sterol accumulation and its implication on child development.

## 1. Introduction

Childhood nutrition is known to play a major role in the onset of chronic diseases later in life. A large body of evidence has convincingly shown that childhood obesity is correlated with risk factors for cardiovascular disease (CVD), metabolic syndrome, and early development of atherosclerosis in adulthood. Few longitudinal studies specifically link childhood obesity to CVD outcomes in adulthood, as it takes decades to evaluate the effect of the gradual process of atherosclerosis [1,2]. Unhealthy childhood nutrition (i.e., low fruit and vegetable intake) are linked not only to an overweight BMI, but to adulthood CVD risk factors and to early vascular changes predicting the risk of CVD [3]. These findings have emphasized the importance of prevention and early management of obesity in the young, including both dietary and physical activity modification, as well as pharmacologic interventions [1,2].

Early-life interventions on cardiovascular health, before the occurrence of clinical manifestations, could help to counteract the global burden of CVDs [4]. The pathological process of CVDs involves atherosclerosis, which is a chronic inflammatory and progressive disease that begins in childhood [5]. Early atheromatous lesions are typically found in the aorta in the first decade of life, while coronary plaque is found in the second decade, and cerebral artery plaques are found in the third or fourth decades [6]. Evidence indicates that risk factor control beginning in youth might delay the development of CVDs, while treating established CVD in adulthood may be counterproductive and ineffective [4]. Evidence that prolonged exposure to low plasma LDL-C levels is associated with markedly greater reduction in CVD risk, compared to current strategies aimed at lowering LDL-C in middle age, has been provided by meta-analysis of Mendelian randomization studies, involving polymorphisms in six distinct genes of cholesterol metabolism, including the *protein convertase subtilisin/kexin type 9* (*PCSK9*) gene [7,8]. In fact, Libby and Everett recently stated that if adulthood LDL-C concentration was continuously maintained similar to those of newborns, the world would probably not face an epidemic of atherosclerosis [9]. 

Therefore, it is plausible that dietary preventive actions in young individuals could be more effective than in older individuals with established atherosclerosis [3]. Dietary strategies for CVD prevention have been proposed by many studies and often suggest the consumption of bioactive compounds such as omega-3 fatty acids, soybean proteins, polyphenols, and plant sterols [10,11]. Clinical trials have consistently shown that the intake of 0.6–3.3 g/day of plant sterols reduces serum levels of LDL-C by about 6–12% [12]. Plant sterols also show additive effects when combined with statin treatment, further lowering total cholesterol and LDL-cholesterol levels [13]. The Adult Treatment Panel III Report on High Blood Cholesterol (2001), strongly recommends the inclusion of plant sterols as a therapeutic dietary option to reduce LDL-C and suggests an intake of 2 g of plant sterol/stanol esters per day [14,15]. In 2010, the FDA authorized a health claim on the relationship between plant sterol/stanol esters and reduced risk of coronary heart disease (CHD) for use on food labels [16]. Despite the increase in commercial food products with added plant sterols, there is currently no recommendation for plant sterol intake for normocholesterolemic healthy children.

Considering that atherosclerosis begins early in life, the chronic intake of foods that are able to reduce LDL-C could be a clinically relevant strategy to reduce CVD in adulthood. The inclusion of plant sterols in the daily diet represent a plausible approach, and yet little is known about the effects of its supplementation during childhood. The present review details the potential of plant sterols to prevent CVDs, but it also highlights controversial findings that raise questions about the implications of widespread use of plant sterols during childhood. 

## 2. Cardiovascular Diseases (CVDs)

In the last decade, CVDs have been shown as the main global cause of death among noncommunicable diseases (NCDs) [17,18]. Between 1990 and 2016, the number of fatal cardiovascular events increased by 53.7% in people over 70 years old. Only in 2016, the total number of deaths from cardiovascular disease was 17.6 million [19]. Recent data also suggest there is a new epidemic of CVD in young populations as consequence of a high-risk lifestyle profile, which includes substance abuse, high BMI, and an elevated rate of diabetes [20].

CVD refers to the group of illnesses that affects heart and blood vessels, including coronary heart disease, high blood pressure, cerebrovascular disease (stroke), heart failure, rheumatic heart disease, congenital heart disease, cardiomyopathies, and peripheral vascular disease [21]. The pathological processes of these illnesses have the progression of atherosclerosis as a common precursor [22]. Atherosclerosis is a chronic inflammatory disorder that produces arterial plaques characterized by inflammatory infiltrate and accumulation of lipids as well as apoptotic and fibrotic cells, leading to arterial stiffness [23].

The biological process of atherosclerosis begins with changes in the inner layer of the artery. Endothelial cells are usually inert to the attachment of leukocytes circulating in the blood; however, irritative stimuli (like dyslipidemia, hypertension, and regions of low shear stress) triggers the expression of adhesion molecules. Increased endothelial permeability and changes in the extracellular matrix promote low-density lipoprotein (LDL) infiltration and retention [23]. Trapped LDL becomes the target for oxidative modifications by reactive species and enzymes, producing damage-associated molecular patterns (DAMPs), which in turn promote monocyte migration [24]. In the sub-endothelium, monocytes maturate into macrophages that phagocytose LDL, secrete cytokines, proteases, or procoagulant factors, and ultimately undergo apoptosis and coalesce into necrotic plaques [25].

The progression of atherosclerosis is related to the anchorage of smooth muscle cells (SMCs) from the media layer. Thereafter, collagen, elastin, and proteoglycans are synthesized and wrap the plaque, causing intimal thickening along with fatty streaks [23]. Over time, additional lipoprotein retention is promoted by elevated extracellular matrix component production, necrotic tissue accumulation, and elevated recruitment of inflammatory cells leading to lesion enlargement [25].

Studies have shown that early arterial lesions, called fatty streaks, are not harmful themselves, but progression into advanced lesions can lead to plaque rupture, causing cardiovascular events such as myocardial infarction [5]. Therefore, even though the clinical manifestation of atherosclerosis is often noticed in midlife or past middle-age, its development begins early in life, as fatty streaks are its precursor. Early development of atherosclerotic plaques is associated with genetic disorders that correspond with increased cardiovascular risk, such as familial hypercholesterolemia (FH) in which raised blood cholesterol is linked to premature ischemic heart disease [26]. Additionally, studies have demonstrated that loss of PCSK9 function derived from gene mutation is associated with cardiovascular protection in individuals whom exposure to LDL was greatly reduced since childhood [23].

The currently available pharmaceutical interventions for cardiovascular disease are designed to slow the progression of atherosclerosis by controlling hypertension and hyperlipidemia [24]. However, data supports inflammatory responses as the major contributor to fatty streak progression [23,24,27,28]. Since fatty streaks are observed in early childhood, it is important to address the viability of its management early in life. As prescribed medications are not an option for healthy children, functional foods, such as plant sterols, are good candidates for alternative intervention. The exception is FH children; they are a special risk group for CVD because of their increased LDL plasma concentrations as a consequence of a dysfunction in LDL receptor (LDLr) endocytic and recycling pathways [29]. For this group, pharmacological intervention with statins starting usually at age 8 is strongly recommended [30,31].

## 3. Plant Sterols

Plant-derived sterols, or phytosterols, are phytochemicals structurally similar to cholesterol, having cellular functions in plants analogous to those of cholesterol in animals and humans [32,33]; that is, they play a major role in regulating membrane fluidity and cellular communication [34].

**Figure 1:**
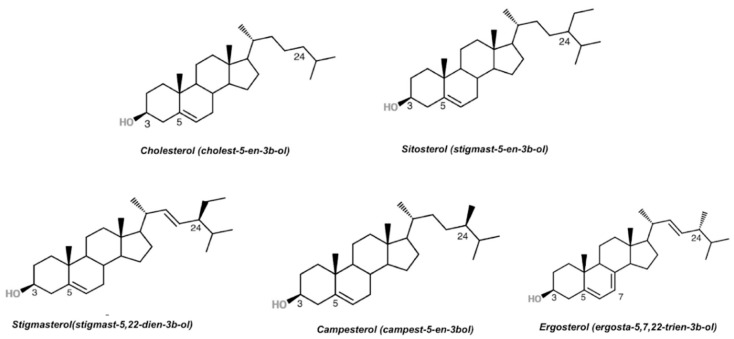
Chemical structures of cholesterol and plant sterols (sitosterol, stigmasterol, campesterol, and ergosterol).

While plant cell membranes have a diverse composition of sterols (e.g., sitosterol, stigmasterol, campesterol, and around 250 more compounds), the predominant sterol found in animal cells is cholesterol [35] (Figure 1). Although plant sterols only slightly differ from cholesterol in the structure of their side chain (by a methyl or an ethyl group at C-24), these phytochemicals are non-nutritive compounds to humans [36].

Plant sterols occur naturally in small amounts in all plant-based foods, most notably in seeds, oilseeds, nuts, cocoa butter, legumes, and grains [37]. Of the various vegetable oils, corn oil, rapeseed oil and wheat germ oil typically have the highest total phytosterol contents [35]. Food products enriched with plant sterols have also been on the market since 1995, pioneered by plant stanol rich margarine. Since then, plant sterols have been added in its esterified form to a wide range of food products such as yoghurts, salad dressings, milk, juice, and snack bars [34,38]. Esterification increases plant sterol lipid solubility in food and is typically performed by adding an 18-carbon fatty acid (e.g., oleic or linoleic) moiety at the C-3 of the sterol structure (‘‘OH position’’) (Figure 1). Commercially produced plant sterol esters are usually obtained from soybean oil [39].

**Figure 2:**
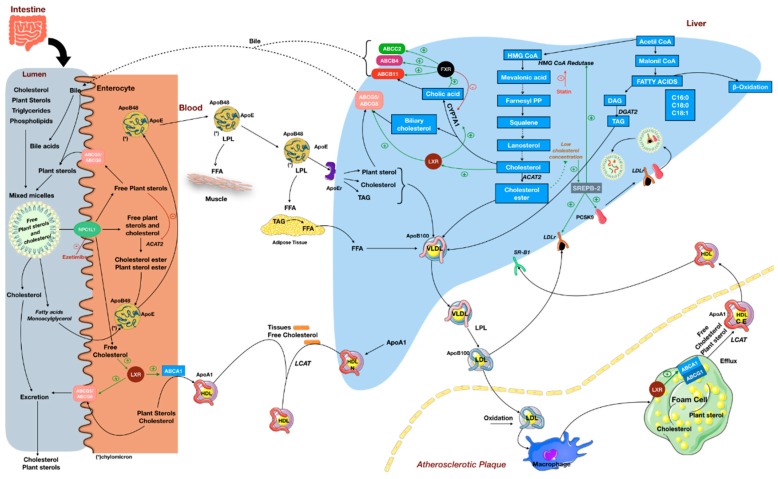
Plant sterol and cholesterol metabolism. Dietary lipids, biliary cholesterol, and bile acids are incorporated into mixed micelles in the intestinal lumen. Competition between plant sterols and free cholesterol during digestion causes a reduction in cholesterol solubilization and increases cholesterol excretion in feces. Free cholesterol and free plant sterols are absorbed through the NPC1L1 transporter, while other lipids are taken up into the enterocyte by facilitated diffusion at the brush border. Plant sterol absorption is controlled by ABCG5/8, which acts as an efflux pump to export free sterols from enterocytes back into the intestinal lumen. Alternatively, plant sterols may also be packed into lipoproteins in a similar way as cholesterol. After intestinal uptake, dietary and biliary free cholesterol (and some free plant sterols) are normally esterified by ACAT-2, incorporated into chylomicrons, and secreted into the lymph. Unesterified cholesterol is secreted back to the intestinal lumen by ABCG5/8. Chylomicrons reach the circulation and deliver free fatty acids to peripheral tissues through the activity of LPL. Chylomicron remnants undergo hepatic uptake, where they contribute to the formation of VLDL along with cholesterol esters (synthesized through the HMG CoA pathway) and TAG (synthesized through the malonyl-CoA pathway). Once plant sterols reach the liver, they can also be returned to the intestine by ABCG5/8 transporters at the hepatobiliary interface. VLDL particles are secreted into the bloodstream and give rise to LDL particles. LDL distributes cholesterol and plant sterols to tissues and undergoes hepatic uptake through LDL receptors (LDLr). However, LDL particles may infiltrate the endothelial intima where they undergo oxidative and enzymatic modification. Uptake of modified LDL by intima macrophages leads to the formation of foam cells and fatty streaks. The atherosclerotic process may be attenuated by HDL, as it promotes cholesterol efflux from other tissues by LCAT, and also from the macrophages. HDL is recognized by SRB1 receptors in the liver and deliver cholesterol (or plant sterols) for biliary excretion, keeping the “cholesterol reverse transport” cycle. Hepatic cholesterol homeostasis is controlled by several sensors. Decreased levels of cholesterol esters activate SREBP-2, which upregulates HMG CoA reductase, increases LDL receptor (LDLr) expression, and induces expression of PCSK9, that after secretion binds to LDLr on the hepatocyte surface, forming a complex PCSK9–LDLr, which is internalized and undergoes degradation. On the other hand, increased levels of cholesterol in hepatocytes leads to activation of LXR and FXR, which upregulate expression of enzymes and transporters involved with biliary excretion. Activation of LXR in enterocytes leads to luminal excretion of cholesterol and facilitates intestinal HDL synthesis. For details on lipid metabolism see Ref. [41,42,43]. Abbreviations: ABCA1, ATP-binding cassette, subfamily A, member 1; ABCG1, ATP-binding cassette, subfamily G, member 1; ABCG5, ATP-binding cassette, subfamily G, member 5; ABCG8, ATP-binding cassette, subfamily G, member 8; ACAT2, acetyl-CoA acetyltransferase 2; CE, cholesterol ester; CM, chylomicron; DAG, diacylglycerol; DGAT2, diacylglycerol O-acyltransferase 2; FFA, free fatty acids; FXR, farnesoid X receptor; HMG Coa-Reductase, 3-hydroxy-3-methyl-glutaryl-coenzyme A reductase; HDL, high-density lipoprotein; LCAT, lecithin-cholesterol acyltransferase; LPL, lipoprotein lipase; LXR, liver X receptor, NPC1L1, Niemann–Pick C1 like 1; PCSK9, proprotein convertase subtilisin/kexin type 9; SBR1, Scavenger receptor class B member 1; SREBP-2, sterol regulatory element-binding transcription factor 2; TAG, triacylglycerol; and VLDL, very low density lipoprotein.

Around 50%–80% of the dietary intake of plant sterols comes from vegetable oils, spreads and margarines, breads, cereals, and vegetables; fruit intake adds a further 12%. While a regular diet provides about 300–400 mg of plant sterols daily, this amount may be two times higher for vegetarians or by the inclusion of plant sterol-enriched food products in the daily diet. The most abundant compounds are sitosterol and campesterol, which contribute 60% and 20%, respectively, of total plant sterol intake [7]. However, the intestinal absorption of plant sterols is very limited in healthy subjects (about 0.5–2%), while absorption efficiency of daily consumption of 250–500 mg of cholesterol is 50–60%. Although plant sterols are taken up from the intestinal lumen into the enterocyte through the same transporter as cholesterol, Niemann-Pick C1-Like 1 (NPC1L1), absorption of these compounds is controlled by ATP-binding cassette co-transporters G5 and G8 (ABCG5/ABCG8), which act as efflux pumps to export free sterols from enterocytes back into the intestinal lumen [40] (Figure 2, [41,42,43]). The few plant sterol molecules that escape from this clearance mechanism are carried by lipoproteins in the circulation in a similar quantitative distribution as cholesterol molecules, therefore circulating primarily in LDL particles (70–80%). The plasmatic levels of plant sterols, however, are around 500- to1000-fold lower than those of cholesterol [7]. Free plant sterol may also be mobilized via ATP-binding cassette transporter A1 (ABCA1) located at the basolateral membrane of enterocytes and becomes a part of high-density lipoprotein (HDL) particles (the same way that cholesterol is mobilized). Once plant sterols reach the liver they can also be returned to the intestine by ABCG5/8 transporters at the hepatobiliary interface [44,45] (Figure 2). 

Nevertheless, when plant sterol intake is high (e.g., with intake of plant sterol-enriched foods), plasma plant sterol concentrations modestly increase. Ras and colleagues (2016) showed that consumption of a low-fat spread with added plants sterols (3 g/day) increases plasma concentrations of sitosterol and campesterol in the first four weeks, while no further increases were observed in the following eight weeks of consumption. However, concentrations at the end of the intervention did not exceed physiological values usually observed in the general population (2.8 to 27.9 µmol/L) [46].

## 4. Cholesterol-Lowering Effect of Plant Sterols and Its Implications

Inhibition of intestinal absorption of cholesterol is the primary action of plant sterols that confers its hypocholesterolemic effect. The most well-documented mechanism described is a dynamic competition between plant sterols and free cholesterol for incorporation into the mixed micelles during the lipid digestion process, which causes a reduction in cholesterol solubilization [47]. As plant sterols are more hydrophobic than cholesterol, the affinity of plant sterols for micelles exceeds that of cholesterol. Subsequently, cholesterol molecules get displaced from mixed micelles, and unabsorbed dietary cholesterol is excreted in feces along with recirculating endogenous biliary cholesterol, in turn reducing absorption and the total pool of cholesterol in the organism [37,48] (Figure 2). 

However, studies have suggested that it is not necessary for plant sterol intake to be a part of the main meal, and they do not need to be present in the intestinal lumen simultaneously with cholesterol to inhibit its absorption [34]. Therefore, other potential mechanisms were proposed. 

After intestinal uptake, dietary and biliary free cholesterol are normally esterified by acylcoenzyme A cholesterol acyltransferase 2 (ACAT-2), incorporated into chylomicrons and then secreted into the lymph. It has been suggested that plant sterols interfere with cholesterol esterification inside the enterocyte, since plant sterols are poor substrates for ACAT-2, and therefore could decrease its activity by competitive inhibition. Unesterified cholesterol is then secreted back to the intestinal lumen by the ABCG5/8 heterodimer transporter, along with free plant sterols. However, the reduced ACAT activity could also be a consequence of decreased cholesterol uptake from the intestinal lumen, as result of a displacement from mixed micelles, once ACAT activity inside the enterocyte may be regulated by substrate supply [49] (Figure 2). A role for ABCG5/8 in facilitating transintestinal cholesterol excretion after plant sterol intake has also been proposed by Brufau et al. (2011) [50]. 

It has also been also proposed that plant sterols may interact with intestinal cholesterol sensors, such as liver X receptor (LXR), contributing to decreased cholesterol absorption [51]. LXR tissue-specific actions are responsible for maintaining systemic cholesterol homeostasis. LXR is induced in response to elevated cellular levels of cholesterol and regulates the expression of genes that encode proteins involved in cholesterol absorption, transport, efflux, excretion, and conversion to bile acids. In the liver, activation of LXR promotes the conversion of cholesterol into bile acids via cytochrome P450 7A1 (CYP7A1) and biliary cholesterol excretion through ABCG5/8. In macrophages, LXR increases the expression of ABCA1 and ABCG1, promoting cholesterol efflux to HDL. In the intestine, LXR activation increases HDL formation via basolateral ABCA1 and promotes transintestinal cholesterol excretion through ABCG5/8 [41] (Figure 2). Recent findings, however, demonstrate that plant sterols reduce plasma cholesterol levels via LXR-independent mechanisms [52]. 

Data comparing the efficacy of single versus multiple daily doses of plant sterols are conflicting [48]. However, the timing of the intake of plant sterols may become more important when a single daily dose is consumed, as it has been documented that a single dose of plant sterols when consumed without a meal may have little effect on LDL-C compared to a single dose (in the same delivery form) consumed with a meal [35]. These observations may be explained by the fact that the oil phase is crucial for the formation of mixed micelles, which subsequently transport the digested and emulsified nutrients and minor food components toward the enterocyte brush border. Therefore, it is of utmost importance that the ingestion of plant sterols, either through supplements or enriched foods, induce bile flow and release of pancreatic lipases during the digestive process [49]. 

While plant sterols decrease the serum non-HDL cholesterol concentration, especially LDL-C, in general their intake does not affect other lipoproteins [38], although a reduction in fasting triglycerides was recently described [53]. The consumption of these compounds is generally described as safe [54], although one potential safety concern is the exposure to plant sterols in the rare inherited disorder called phytosterolemia (or sitosterolemia). This condition is caused by loss of function mutations in the ABCG5/8 heterodimer transporter, both in intestine and in liver, which leads to partial or complete failure of sterol efflux and consequently plant sterol accumulation in plasma and tissues [32]. Development of xanthomas, down-regulated cholesterol biosynthesis, along with increased expression of low-density lipoprotein receptor (LDLr) and sometimes premature atherosclerosis, underpins the etiology of this disease [44]. Patients with sitosterolemia may also show arthralgia, thrombocytopenia, and hemolysis probably because of the incorporation of plant sterols into the red blood cell membrane [55].

Absorption of dietary plant sterols is increased by 40–60% in phytosterolemic patients, approaching the absorption of cholesterol, in contrast to the typical <5% absorption rate in normal individuals. Plasma plant sterol levels in this condition ranges from 30 to 60 mg/dL. Some phytosterolemic patients also present with pseudo-homozygous FH, which is due to a complete failure of cholesterol efflux into bile [44,56]. Both elevated plasma cholesterol and plant sterol levels in phytosterolemia may contribute to premature vascular disease in early childhood or later in life [44], as it has been suggested that abnormal cholesterol metabolism likely contributes most strongly to the resulting atherosclerosis than plant sterol accumulation itself [32,57]. In a recent study including five phytosterolemic subjects, Hansel et al. showed that, in spite of massive hypercholesterolemia and high plant sterol levels, none of the subjects had clinical symptoms of CVD or positive clinical markers of atherosclerosis [38,58]. Although there are only 80–100 cases of known phytosterolemia in the world, Mymin et al. recently suggested that the prevalence is greater than previously suspected, and true morbidity rate is low, because a milder phenotype without specific symptoms exists [59].

The levels of plant sterols in plasma, taken together with cholesterol precursor sterols, can also serve as surrogate markers of cholesterol absorption and metabolism. Under normal conditions, the homeostasis of whole-body cholesterol is mainly controlled by cholesterol synthesis and absorption. These pathways are interdependent, meaning that the synthesis of cholesterol is influenced via complex feedback mechanisms by the amount of dietary cholesterol and cellular requirements and vice versa [55]. Individuals with lower plasma markers of cholesterol synthesis (i.e., cholesterol precursor sterols cholestenol, desmosterol, and lathosterol) and higher markers of cholesterol absorption (plant sterols, campesterol, sitosterol) are “hyperabsorbers” of cholesterol. These subjects tend to exhibit greater LDL-C lowering in response to phytosterol supplementation and cholesterol absorption inhibitors such as ezetimibe [60,61,62]. In fact, cholesterol absorption follows a typical Gaussian distribution: while the average person absorbs about 55% of cholesterol from normal dietary intake, a small proportion of the population hyperabsorbs 90% of dietary cholesterol or underabsorbs less than 10% [63].

## 5. Evidence from Clinical Trials

The LDL-cholesterol-lowering effect of plant sterols or stanols (the 5α-saturated derivatives of plant sterols) have been repeatedly confirmed by more than 200 studies (Table 1 summarizes some of the studies). The efficacy of the LDL-C-lowering effect, however, is affected by the subject’s baseline cholesterol concentration, the food matrix, frequency and time of intake, and dosage [12,64,65]. According to these data, a 2 g/day threshold dose of plant sterols/stanols is generally required for a clinically relevant LDL-C reduction. However, to achieve that, humans living in the developed world are expected to ingest plant sterol-enriched foods or supplements [48]. 

Large meta-analyses have indicated that plant sterols and stanols work equivalently in clinical trials, both leading to inhibition of cholesterol absorption [48,66]. Demonty et al. (2009) evaluated 165 clinical trials based on the following criteria: randomized controlled trials with adults treated with plant sterols (4-desmethylsterols and/or 4-desmethylstanols extracted from vegetable or plant oils) and absence of a co-intervention from which consumption of plant sterol-enriched foods or supplements could not be isolated. Their main result is the observation of a continuous dose-response relationship for the LDL-C–lowering effect and plant sterol intake associated with a plateau when it achieves approximately 3 g/day, corresponding to an LDL-C lowering around 10.7% [66]. Gylling et al. (2013) published a controlled randomized trial in which 92 asymptomatic subjects ingested 3 g of plant stanols daily through rapeseed oil-based enriched spread for 6 months. Results showed an LDL-C decrease of 10% and reduction of arterial stiffness in small arteries—a marker of subclinical atherosclerosis (cardio-ankle vascular index—CAVI) [67]. 

Numerous trials suggest that consumption of plant sterols supplements and enriched foods would lower the risks of CVD based on its effect on LDL-C [68,69,70]. However, clinical trials demonstrating potential effects of plant sterol supplementation on hard clinical endpoints of atherosclerosis are still lacking. Despite plasma cholesterol reduction by plant sterol intake, it is not possible to conclude that decreased plasma LDL-C automatically prevents atherosclerosis progression, and consequently CVD risk, as atherosclerosis is multifactorial. Controversially, the PROCAM trial [71] demonstrated an association between elevated concentrations of sitosterol and increased incidence of coronary events in men at high cardiovascular risk. Moreover, other studies including different populations have found similar results [72,73,74]. On the other hand, Wilund et al. (2004) and Pinedo et al. (2007) showed that dietary plant sterol supplementation is unlikely to confer an increased risk of atherosclerosis in adults [75,76]. The effect of long-term consumption of plant sterols by children, however, is even less clear. 

## 6. Plant Sterol Supplementation in Childhood

Although childhood plant sterol supplementation is generally recognized as safe during short-term periods, the most recent NCEP guidelines only recommends the intake of plants sterols/stanols for children with FH after 2 years of age [84]. The European Atherosclerosis Society Consensus Panel on Phytosterols, however, does not advise the use of functional foods enriched with plant sterols by any children under 6 years [7]. The rationale for such recommendations is not entirely clear. The efficacy of plant sterols to reduce LDL-C during youth has been shown by a considerable number of studies, when both normolipidemic and hyperlipidemic children aged 4–19 years receiving 1.2–3 g/day of plant sterols/stanols showed the same rate of LDL-C reduction as seen in adults (5%–20%) [7,77,78,79,80,81,82,83,85,86].

Despite reductions in total and LDL-C, the intake of plant sterols and stanols by prepubertal FH children did not improve endothelial function in two previous studies, and this led authors to suggest that raised sterol plasma concentrations may negatively affect endothelial function, counteracting the beneficial effects of plant sterols on cholesterol levels [81,82]. In a previous study carried out by our group, mice that were supplemented with plant sterols during the first 4 months of life showed increased atherosclerotic lesion areas after receiving a high-fat diet for 8 weeks, compared to a control group on high-fat diet supplemented with soybean oil [87]. This unexpected result raised concerns and questions on the difference between plant sterol metabolism in children and adults. 

An age-dependent effect on sterol esterification was already described for plasma cholesterol, with infants showing lower lecithin cholesterol acyltransferase (LCAT) activity than adults [88,89]. Recent data also showed that preterm infants receiving parenteral nutrition (PN) have markedly decreased plant sterol esterification compared to adults receiving PN, which was supposedly related to lower LCAT activity [90]. LCAT is a key enzyme for reverse cholesterol transport, as it esterifies free cholesterol at the surface of the HDL particle, after which cholesterol esters migrate to the particle core. Impaired LCAT activity has been associated with premature coronary artery disease [91,92].

Lipid emulsions containing plant sterols have also been implicated with cholestasis in infants receiving PN [93,94,95]. During PN, the enterohepatic circulation of bile acids is disrupted because of the absence of intestinal flow, and bile acids accumulate in the liver. Plant sterols present in soybean oil (SO) lipid emulsions may aggravate this condition by binding to sterol carrier proteins and impairing the movement of cholesterol, bile acid precursors, and other lipids across the cell. Furthermore, it has been suggested that major plant sterols could lead to substitution of cholesterol in liver cell membranes, interfering with membrane function [93,96]. More recently, a mechanistic study has shown that stigmasterol, one of the three predominant plant sterols in SO emulsions, is a potent inhibitor of farnesoid X receptor (FXR) activity in cultured hepatocytes and, therefore, impairs the transcription of bile acid transporters [97] (Figure 2). The effects of plant sterols in the suppression of bile acid, bilirubin, and sterol transporters was further corroborated by Kasmi et al. [98]. These authors showed that gene expressions of the canalicular bile acid transporter *Abcb11*, conjugated bilirubin transporter *Abcc2,* and *Fxr* were reduced in mice that were infused with plant sterol-containing emulsions, but not in mice receiving plant sterol-free PN, and that serum stigmasterol concentrations correlated with the severity of cholestasis. Mice receiving intravenous stigmasterol also had markedly reduced gene expressions of *Abcg5, Abcg8*, and *Lxr,* which further contributes to plant sterol accumulation [98].

Unlike plant sterol supplementation through fortified food, intravenous administration of SO lipid bypasses protective mechanisms that prevent against phytosterol accumulation in the body [99]. However, it has been suggested that age might be associated with toxic effects of plant sterols. It has been shown that plant sterols rapidly accumulate in neonates during SO lipid infusions, especially in very preterm infants, which suggests they may have poorly developed mechanisms of metabolizing plant sterols compared to full-term infants [99]. After prolonged PN, higher plant sterol levels were also seen in neonates compared to older children [100]. Absorption of plant sterol seems to decrease with age, as studies have shown that infants given a plant sterol-rich diet had higher absorption than children [101], and absorptions seen in infants and children were both shown to be higher than those observed in adults [99,102].

Almost half a century ago, Mellies et al. shed light on the importance of monitoring the unanticipated, long-term effects of cholesterol-poor, plant sterol-rich diets in infants and children [101]. Dietary recommendations for children and adolescents have suggested the replacement of saturated fat in the diet with monounsaturated (MUFA) and polyunsaturated fatty acids (PUFA) as an approach to prevent CVDs [103]. This modification, on the other hand, lead to an increased intake of vegetable oil, and consequently, plant sterols. The introduction of infant formulas, for example, contributed to increase plant sterol consumption once they contain higher amounts of plant sterols than human milk [104]. Three- to fivefold increases in plasma plant sterols (campesterol, stigmasterol, and β-sitosterol) have been reported in children on cholesterol-lowering diets and in infants taking commercial formulas (rich in vegetable oil) but not in adults who consume such a fat-modified diet [101]. Replacement of milk fat, which is rich in saturated fat but contains no plant sterols, with low erucic acid rapeseed oil, which is rich in MUFA and plant sterols, from the age of 12 months was shown to promote a twofold intake of plant sterols compared to children consuming conventional diets [85]. Although the implications of plant sterol plasma elevations over time is currently unknown, plant sterols were shown to accumulate in normal aortic tissue of infants on phytosterol-rich diets [105]. Plant sterol-rich infant formulas are also suggested to increase endogenous cholesterol synthesis in neonates [106,107].

Recently, Mymin and collaborators (2018) [59] showed that an age-related change in cholesterol and sterol homeostasis occurs at puberty in phytosterolemia. Their data suggest the existence of an inverse relation between age and plasma levels of cholesterol and sitosterol, and there is an age threshold, at or about puberty, when levels of sitosterol and cholesterol spontaneously decrease. This change is thought to be due to high sterol and/or stanol levels causing feedback inhibition of sterol regulatory element-binding protein (SREBP-2) processing (Figure 2). This would explain the well-documented phenomenon of depressed cholesterol synthesis in phytosterolemia. It is also well-known that LDL-receptor activity is increased during phytosterolemia, and this could feasibly explain the decrease in LDL cholesterol and consequent reduction of plasma total cholesterol and sitosterol levels observed at puberty. Downregulated SREBP-2 processing would be expected to also lower PCSK9 levels, which would explain high LDL-receptor activity [59] (Figure 2). Changes in cholesterol homeostasis around puberty were also documented in healthy children/adolescents, with increased cholesterol absorption being reported at ages below 10 years, after which cholesterol synthesis prevails [108]. Data from the study also show a twofold increase in serum campesterol from the age of 1 year, which did not occur for sitosterol—the main dietary plant sterol. Whether the noncholesterol sterol absorption rate is increased remains to be elucidated [108].

However, the implications for accumulated phytosterols are currently not fully understood, and long-term safety studies are necessary.

## 7. Conclusions

Plant sterol supplementation is undoubtedly effective at lowering LDL-C, the main risk factor for atherosclerotic disease. Conflicting data and the lack of well-defined endpoint to clinical studies, however, contribute to skepticism regarding plant sterols’ ability to reduce cardiovascular events. Notwithstanding, it is still not established if plant sterols could impede atherosclerosis progression or attenuate the consequences of the disease.

Although we agree that cardiovascular disease prevention should start as early as possible, maturation of biologic process should be considered before any dietary supplementation. Replacing cholesterol/animal fats with vegetable oil during infancy and childhood should be carefully evaluated. There is a gap in the knowledge of whether increased absorption as well as decreased esterification of plant sterols occurs during infancy and childhood, which would negatively impact hepatic function and reverse transport of plant sterols from peripheral tissues (and atherosclerotic plaque macrophages) to the liver. Considering all these arguments, any recommendation for widespread use of plant sterols in children for cardiovascular prevention has yet to be substantiated. More studies are needed to elucidate plant sterol metabolism during infancy and childhood and the long-term effects of plant sterol accumulation.

## Figures and Tables

**Table 1:** Summary of studies.

Reference	Study Type	Objectives	Main Results
Demonty et al. (2009) [66]	Meta-analysis of randomized controlled trials in adults treated with plant sterols without a co-intervention. Consumption of plant sterol-enriched foods or supplements could not be isolated	Establish a continuous dose–response relationship that would allow predicting the LDL-C-lowering efficacy of different plant sterol doses.	The dose–response equation predicts an LDL-C-lowering effect of 9% for the recommended 2 g/day dose of plant sterols. The continuous dose–response relationship for the LDL-C-lowering effect and plant sterol intake achieved a plateau when it came to approximately 3 g/day.
Gylling et al. (2013) [67]	Randomized, controlled, double-blind, parallel trial including 92 asymptomatic subjects (35 men and 57 women, mean age of 50.8 ± 1.0). The subjects consumed 3 g of plant stanols daily through rapeseed oil-based enriched spread for 6 months.	Evaluate the effects of plant stanol esters on arterial stiffness and endothelial function in adults without lipid medication.	LDL-C decrease of 10% and reduction of arterial stiffness in small arteries and marker of subclinical atherosclerosis (cardio-ankle vascular index—CAVI)
Ras et al. (2014) [12]	Meta-analysis of randomized controlled studies in adults. In total, 124 human studies with a total of 201 study arms were included. Plant sterols and stanols were administered in 129 and 59 study arms, respectively; in the remaining 13 study arms, a mix of plant sterols and stanols was administered.	To investigate the combined and isolated effects of plant sterols and stanols by evaluating different dose ranges.	The average phytosterol (comprising plant sterols and plant stanols) dose 2.1–3.3 g/day were found to gradually reduce LDL-C concentrations by 6%–12%.
Matvienko et al. (2002) [68]	Triple-blind, 34 male college students with elevated total plasma cholesterol (TC), LDL-C, and TC:HDL-C. Randomized: control (ground beef alone) or treatment (ground beef with 2.7 g of plant sterols) group.	Test the hypothesis that a single daily dose of soybean plant sterols added to ground beef would lower TC and LDL-C concentrations in mildly hypercholesterolemic young men.	TC, LDL-C, and TC:HDL-C were reduced from baseline by 9.3%, 14.6%, and 9.1%, respectively.
Assmann et al. (2006) [71]	Case–control study using stored samples from male participants in the Prospective Cardiovascular Münster (PROCAM)	Evaluate if modest sitosterol elevations observed in the general population is associated with the occurrence of coronary events.	Among men with an absolute coronary risk ≥20% in 10 years, high sitosterol concentrations were associated with an additional 3-fold increase in the incidence of coronary events; a similar, significant relationship was observed between a high sitosterol/cholesterol ratio and coronary risk
Mussner et al. (2002) [69]	Randomized, double-blind, placebo-controlled, cross-over study including 63 healthy subjects (38 women, 25 men, mean age of 42 years old, LDL-C of 130 mg/dL)	Comparison of effects from the intake of a plant sterol-enriched margarine and a control margarine.	Plant sterol ester-enriched margarine significantly changed TC, LDL-C HDL-C, apolipoprotein B, and the LDL-C/HDL-C ratio compared to the control margarine
Wilund et al. (2004) [75]	Human subjects from the Dallas Heart Study, 2542 subjects aged 30 to 67 years, were included. Wild-type hypercholesterolemic female mice were also studied.	Determine whether elevated plasma levels of plant sterols were associated with coronary atherosclerosis humans and mice.	Plasma levels of cholesterol, but not of plant sterols, were significantly higher in subjects with coronary atherosclerosis.
Pinedo et al. (2007) [76]	Case–control study among participants of the EPIC-Norfolk Study. Only individuals who did not report a history of heart attack or stroke at the baseline clinic visit were considered.	Evaluate the relationship between plant sterol levels and coronary artery disease risk	Higher levels of plant sterols are unlikely to confer increased risk of coronary artery disease in healthy adults.
Williams et al. (1999) [77]	Open cross-over randomized study lasting 13 weeks; eligible children started either with the diet phase A (plant stanol ester) or B (wheat bran fiber). The first diet phase lasted 4 weeks, and then they went under a two-week wash-out followed by a cross-over to the other diet for 4 weeks.	Evaluate the effects of plant stanol ester in healthy two- to five-year-old preschool children.	Reductions in TC and in LDL-C by 12.4% and 15.5%, respectively, from baseline were observed. There were no significant changes in HDL-C or triglyceride levels.
Guardamagna et al. (2011) [78]	Interventional study using plant sterol-enriched yoghurt for 12 weeks in 32 children with heterozygous familial hypercholesterolemia (FH), 13 children with familial combined hyperlipidemia (FCH), and 13 children with undefined hypercholesterolemia (UH).	To access the efficacy, tolerability, and safety of plant sterol supplementation in children with primary hyperlipidemia.	LDL-C was significantly reduced in the three groups of different forms of primary hyperlipidemia (10.7%, 14.2%, and 16.0% in FH, FCH, and UH, respectively). High tolerability to the diet was observed.
Becker et al. (1993) [79]	Interventional study in 9 children with severe familial hypercholesterolemia. Firstly, there was a 3-month strict diet, followed by the intake sitosterol pastilles (2 g three times a day) for 3 months, and then a 7-month course of sitostanol (0.5 g three times a day).	Set the efficacy difference between sitostanol, a nonabsorbable plant sterol, and sitosterol to reduce serum levels of lipids in children with severe familial hypercholesterolemia.	Sitostanol was significantly more effective in reducing elevated levels of LDL-C than sitosterol (32%).
Amundsen et al. (2002) [80]	Randomized, double-blind crossover study with 38 children (aged 7–12 years) with familial hypercholesterolemia (FH) consuming plant sterol ester enriched spread or a control spread.	Access the effects of plant sterol ester enriched spread intake on serum lipids in children with FH.	Compared to the control group, a consumption of 1.6 g of plant sterol esters promoted a 10.2% reduction in LDL-C concentrations.
de Jongh et al. (2003) [81]	Double-blind crossover trial using plant sterol enriched spreads and a placebo spread. Forty-one children (aged 5–12 years) with familial hypercholesterolemia (FH) were included in this study.	Evaluate the effect of plant sterols on cholesterol levels and vascular function in prepubertal children with FH.	Compared to the placebo group, the intake of 2.3 g plant sterols per day decreased 11% of TC and 14% of LDL-C.
Jakulj et al. (2006) [82]	Double-blind crossover trial testing low-fat yogurt enriched with plant stanols and low-fat placebo yogurt for 4 weeks. The study enrolled 42 prepubertal children with familial hypercholesterolemia (FH).	Evaluate the effects of plant stanols on lipids and endothelial function in prepubertal children with FH.	The group that consumed plant stanols showed a reduction of 9.2% in LDL-C levels without changes in endothelial function.
Ribas et al. (2017) [83]	Randomized, double-blind, cross-over trial using phytosterol-enriched milk and skim milk. Twenty-eight dyslipidemic children (aged 6-9 years) were included in this study.	Investigate the effects of daily consumption of a phytosterol-enriched milk on the lipid profiles of children with dyslipidemia.	The concentrations of TC and LDL-C were significantly reduced in the phytosterol-enriched milk group as compared to the skim milk group, with reductions of 5.9% and 10.2%, respectively.

## Abbreviations

ABCA1	ATP-binding cassette, subfamily A, member 1
*Abcb11*	ATP-binding cassette subfamily B member 11 gene
*Abcc2*	ATP-binding cassette subfamily C member 2 gene
ABCG1	ATP-binding cassette, subfamily G, member 1
ABCG5	ATP-binding cassette, subfamily G, member 5
ABCG8	ATP-binding cassette, subfamily G, member 8
ACAT2	acetyl-CoA acetyltransferase 2
AHA	American Heart Association
CHD	coronary heart disease
CM	Chylomicron
CVD	cardiovascular disease
DAG	Diacylglycerol
DAMPs	damage-associated molecular patterns
DGAT2	diacylglycerol O-acyltransferase 2
ESC	European Society of Cardiology
FDA	Food Drug Administration
FFA	free fatty acids
FH	familial hypercholesterolemia
FXR	farnesoid X receptor
HMG CoA-Reductase	3-hydroxy-3-methyl-glutaryl-coenzyme A reductase
HDL	high-density lipoprotein
LCAT	lecithin-cholesterol acyltransferase
LDL	low-density lipoprotein
LDL-C	LDL cholesterol
LPL	lipoprotein lipase
LXR	liver X receptor
MUFA	monounsaturated fatty acids
NCD	noncommunicable diseases
NCEP	National Cholesterol Education Program
NPC1L1	Niemann–Pick C1 Like 1
PCSK9	proprotein convertase subtilisin/kexin type 9
PN	parenteral nutrition; PRRs
PUFA	polyunsaturated fatty acids
SBR1	scavenger receptor class B member 1
SMCs	smooth muscle cells
SO	soybean oil
SREBP-2	sterol regulatory element-binding transcription factor 2
TAG	Triacylglycerol
VLDL	very low density lipoprotein
WHO	World Health Organization
